# Complete Genome Sequence of a Novel Avian Polyomavirus Isolated from Gouldian Finch

**DOI:** 10.1128/genomeA.01001-15

**Published:** 2015-09-17

**Authors:** Kristin Heenemann, Michael Sieg, Antje Rueckner, Thomas W. Vahlenkamp

**Affiliations:** Veterinary Faculty, Center for Infectious Diseases, University of Leipzig, Institute of Virology, Leipzig, Germany

## Abstract

A novel polyomavirus was identified in a fatally diseased Gouldian finch (*Erythrura gouldiae*). The new polyomavirus, strain VL 1209, was detected using a broad-spectrum nested PCR.

## GENOME ANNOUNCEMENT

Polyomaviruses are small, double-stranded, circular DNA viruses with a genome size of about 5,000 bp. Viruses belonging to the family of *Polyomaviridae* are detected in mammalian species, birds, and fish (1). So far, seven different viruses, budgerigar fledgling disease polyomavirus (2), goose hemorrhagic polyomavirus (3), finch polyomavirus (4), crow polyomavirus (4), canary polyomavirus (5), butcherbird polyomavirus (6) and Adélie penguin polyomavirus (7), have been identified in birds. In contrast to the mammalian polyomaviruses, the avian polyomaviruses can cause acute disease with high mortality rates in infected birds (8).

Here we report the complete genome sequence of a new avian polyomavirus. The viral infection was detected in the liver of a Gouldian finch (*Erythrura gouldiae*) with a suspected polyomavirus infection. DNA was extracted and investigated with a broad-spectrum polyomavirus nested PCR (9). The PCR product was submitted to sequencing. On the basis of the sequence, inverse primers were constructed. Viral DNA was amplified using the primed rolling-circle amplification (RCA) method. The RCA product was used as the template for whole-genome amplification, purified, and cloned into pJET1.2/blunt. Sequencing was done by using the primer walking method with dideoxy Sanger technology and were assembled based on overlapping regions.

The genome of the avian polyomavirus consists of 5,172 bp with an overall GC content of 44.59%. Like other avian polyomaviruses, the genome is composed of an untranslated regulatory region flanked by the genes of the large and small T antigen and the genes of the structural proteins VP1, VP2, VP3, and open reading frame (ORF)-X. A BlastX search of the complete nucleotide sequence revealed the highest homology (71%) to the crow polyomavirus (accession number DQ192570). Except ORF-X, the amino acid identity of all genes showed highest homologies (59% to 81%) to the crow polyomavirus. ORF-X displayed the highest amino acid homology of 41% to the butcherbird polyomavirus. In the novel polyomavirus, the highly conserved Gxxx-VNLE motif, GAVP-VNLE, was detected in the large T antigen.

The obtained data will be helpful for further investigations and diagnostic purposes on polyomavirus infections in avian species.

### Nucleotide sequence accession number.

This whole-genome project has been deposited in GenBank under the accession number KT302407. The version described in this paper is the first version.
